# Wohlbefinden bei Patienten mit Vitiligo mit Beteiligung der sichtbaren Hautareale und der Genitalregion in Deutschland– eine fragebogengestützte Pilotstudie

**DOI:** 10.1111/ddg.15891_g

**Published:** 2026-02-05

**Authors:** Janne Ohlenbusch, Rachel Sommer, Kerstin Steinbrink, Markus Böhm

**Affiliations:** ^1^ Klinik für Hautkrankheiten Universität Münster; ^2^ Institut für Versorgungsforschung in der Dermatologie und bei Pflegeberufen Universitätsklinikum Hamburg‐Eppendorf, Hamburg

**Keywords:** Krankheitslast, psychosoziale Auswirkungen, Lebensqualität, Fragebogenstudie, Burden of disease, psychosocial impact, quality of life, questionnaire study

## Abstract

**Hintergrund:**

Bislang existieren nur wenige Studien zu den psychosozialen Auswirkungen von Vitiligo bei deutschen Patienten.

**Patienten und Methodik:**

Ziel dieser monozentrischen Pilotstudie war es, das Wohlbefinden und die Lebensqualität erwachsener Patienten mit Vitiligo zu erfassen und diese Parameter hinsichtlich Geschlecht, Alter und klinischer Merkmale zwischen Betroffenen mit und ohne Beteiligung des Genitalbereichs, Gesichts und der Hände zu vergleichen. Wohlbefinden wurde mit Hilfe des WHO‐5‐Fragebogens und Lebensqualität mit dem *Dermatology Life Quality Index* (DLQI) ermittelt. Die psychometrischen Eigenschaften des WHO‐5‐Fragebogens wurden in dieser Stichprobe ebenfalls getestet.

**Ergebnisse:**

Insgesamt wurden n = 110 Patienten (mittleres Alter ± SD: 47,5 ± 14,7 Jahre; 54,5% weiblich; 95,5% mit nichtsegmentaler Vitiligo) eingeschlossen. Der Mittelwert des WHO‐5‐Score lag bei 13,3, wobei 42,2% (n = 46) einen Wert von < 13 aufwiesen, was ein vermindertes Wohlbefinden impliziert. 14,7% (n = 16) der Patienten hatten einen WHO‐5‐Score von < 7, was auf eine hohe Wahrscheinlichkeit für eine Depression hinweist. Es wurde kein signifikanter Unterschied zwischen dem WHO‐5‐Score, dem DLQI‐Score und Patienten mit und ohne Genital‐, Gesichts‐ und Handbeteiligung festgestellt. Die psychometrischen Eigenschaften des WHO‐5‐Fragebogens waren gut.

**Schlussfolgerungen:**

Unsere Ergebnisse unterstreichen die psychosozialen Auswirkungen von Vitiligo bei deutschen Patienten. Zudem legen sie die Nützlichkeit des WHO‐5‐Fragebogens als einfaches psychosoziales Screening‐Instrument für die Routineversorgung dieser Patienten nahe.

## EINLEITUNG

Vitiligo ist eine häufige Hauterkrankung mit einer geschätzten weltweiten Prävalenz von 1,1% (bestätigte Diagnose) und 1,3% (unbestätigte Diagnose) basierend auf einer kürzlich durchgeführten Fragebogenstudie in den USA, Europa (einschließlich Deutschland) und Japan.^1^ Vitiligo wird durch eine autoimmunvermittelte Zerstörung der Melanozyten verursacht, die zu weißen Flecken führt. Die nichtsegmentale Vitiligo (NSV), die die Haut symmetrisch betrifft, ist hierbei der häufigste Subtyp und betrifft häufig sichtbare Hautareale wie Gesicht, Hals und dorsale Aspekte der Hände und Finger.^2^ Bislang gibt es nur wenige epidemiologische Daten zu den psychosozialen Auswirkungen von Vitiligo bei deutschen Patienten, wie in einem aktuellen Positionspapier betont wurde.^3^ Ein Befall der Vitiligo in sichtbaren Hautarealen sowie das weibliche Geschlecht wurden vor einiger Zeit mit Stigmatisierung in Verbindung gebracht.^4^ In einer umfangreichen älteren Studie aus dem Jahr 2009 mit 3319 Patienten aus zwei deutschen Vitiligo‐Selbsthilfeverbänden lag der Mittelwert des *Dermatology Life Quality Index* (DLQI), dem am häufigsten verwendeten *Patient‐Reported Outcome Measure* (PROM) für chronische Hauterkrankungen, bei 7,0. In dieser Studie wurden insgesamt 1023 Fragebögen ausgewertet.^5^ Angesichts der zunehmenden Evidenz für eine beeinträchtigte Lebensqualität (QoL), Krankheitslast, Stigmatisierung und psychosoziale Komorbidität bei Patienten mit Vitiligo – wie zahlreiche klinische Studien weltweit zeigen⁶ – betonen wir den dringenden Bedarf an neuen und zusätzlichen Studien in Deutschland zu diesem speziellen Thema sowie die Notwendigkeit einer routinemäßigen Bewertung der psychosozialen Komorbidität.^3^ Kürzlich wurde für Patienten mit nicht‐segmentaler Vitiligo (NSV) und Gesichtsbeteiligung der Januskinase‐(JAK)‐1/2‐Hemmer Ruxolitinib als erste offiziell zugelassene topische Behandlung eingeführt. Ein Befall anderer sichtbarer Areale wie der Hände, aber auch des Genitalbereichs, kann jedoch ebenfalls mit Beeinträchtigungen der Lebensqualität und des Wohlbefindens einhergehen. Aufgrund von Schamgefühl und Unbehagen wird aber eine Beteiligung des Genitalbereichs bei der Vitiligo häufig nicht thematisiert. In dieser monozentrischen Querschnitts‐Pilotstudie haben wir den Einfluss der Vitiligo auf das gesundheitsbezogene Wohlbefinden im Allgemeinen und speziell im Vergleich zwischen Patienten mit und ohne genitale sowie sichtbare (Gesichts‐ und Hand‐)Beteiligung untersucht. Darüber hinaus wurden die psychometrischen Eigenschaften des WHO‐5‐Fragebogens für Vitiligo getestet.

## PATIENTEN UND METHODIK

### Studiendesign

Es handelt sich um eine Querschnitts‐Pilotstudie unter Routinebedingungen bei Patienten mit Vitiligo ab 18 Jahren. Die Genehmigung wurde von der Ethikkommission der Ärztekammer Westfalen‐Lippe eingeholt (2022‐384‐f‐S).

### Patienten

Erwachsene Patienten mit dermatologischer Diagnose einer Vitiligo, die sich einer Routinebehandlung in der Ambulanz der Klinik für Dermatologie der Westfälischen Wilhelms‐Universität Münster (WWU) unterzogen, wurden zwischen Januar 2018 und November 2023 unter Verwendung des Berichtsgenerators und des Codes L80.0 der Internationalen statistischen Klassifikation der Krankheiten und verwandter Gesundheitsprobleme (ICD‐10‐GM) aus dem elektronischen Patientenaktendatensystem (Orbis) abgerufen. Dazu gehörten ausschließlich gesetzlich Versicherte. Patienten, die sich auf Grund anderer Diagnosen stationär in Behandlung befanden, wurden ausgeschlossen. Eingeschlossen wurden nur Patienten mit genau klassifizierter Vitiligo (NSV, segmentale Vitiligo [SV], nicht bestimmt), vollständiger Beschreibung der Krankengeschichte und der betroffenen Körperstellen. Den ausgewählten Patienten wurden sodann die unten aufgeführten Fragebögen sowie ein Informations‐ und Einverständnisschreiben zugesandt. Allen Personen wurde ein dritter standardisierter Fragebogen zur Selbsteinschätzung ihres Hautphototyps zur Verfügung gestellt. Dieser Fragebogen ist beim Bundesamt für Strahlenschutz frei erhältlich (https://www.bfs.de/DE/themen/opt/uv/wirkung/hauttypen/hauttypen.html). Die Datenanalyse erfolgte nach Pseudonymisierung.

### Parameter des Ergebnisses

Zwei standardisierte Fragebögen wurden von den Patienten ausgefüllt:

‐ Die deutsche Fassung der WHO‐5‐Fragebogens.^7^ Dieser zählt zu den generischen globalen Bewertungsskalen zur Messung des subjektiven Wohlbefindens (https://www.who.int‐/publications/m/item/WHO‐UCN‐MSD‐MHE‐2024.01). Der Befragte wird gebeten zu bewerten, wie gut jeder der fünf positiv formulierten Aussagen auf ihn zutrifft, wenn er die letzten 14 Tage betrachtet. Jedes der fünf Itens wird von 5 (die ganze Zeit) bis 0 (zu keinem Zeitpunkt) bewertet. Der Gesamtwert reicht daher theoretisch von 0 (Abwesenheit von Wohlbefinden) bis 25 (maximales Wohlbefinden).
‐ Die DLQI.^8^ Der DLQI ist ein hautgenerischer QoL‐Fragebogen, der zehn Items umfasst, die auf einer 4‐stufigen Likert‐Antwortskala von 0 (überhaupt nicht) bis 3 (sehr) zu beantworten sind. Anschließend wird ein Gesamtsummenwert von 0 bis 30 berechnet, wobei höhere Werte auf größere Beeinträchtigungen hindeuten. DLQI‐Score von > 10 werden als große/extrem große Beeinträchtigungen des Lebens der Patienten angesehen.^9^
‐ Neben Alter und Geschlecht wurden auch klinische Variablen (Art der Vitiligo, Körperoberfläche [body surface area; BSA], Krankheitsdauer, betroffene Hautareale und Begleiterkrankungen) aus der elektronischen Akte und dem medizinischen Bericht des Patienten ermittelt. Diese Variablen wurden vom behandelnden Klinikarzt generiert. Die Auswahl des Hautphototyps erfolgte mittels Fragebogen durch den Patienten, wie oben erwähnt.

### Statistische Analyse

Die Analyse der Daten erfolgte mittels SPSS (Statistical Package für Social Sciences, Version 28) und Microsoft Excel (Version 16.87), wobei der kritische p‐Wert (α) = 0,05 als Signifikanzniveau festgelegt wurde. Deskriptive Statistik (absolute [n] und relative Häufigkeiten [%] für kategoriale Variablen; Mittelwerte und Standardabweichungen [M ± SD] für stetige Variablen) wurden für Alter, Geschlecht und klinische Variablen als auch für den WHO‐5‐ und den DLQI‐Score erstellt. Die Homogenität von Alter, Geschlecht und klinischen Merkmalen der Stichprobe zwischen den Gruppen von Patienten mit und ohne genitale Läsionen wurde durch unabhängige Stichproben, t‐Tests (kontinuierliche Variablen) oder Chi‐Quadrat‐Tests (kategoriale Variablen) untersucht. Um die Lebensqualität und das Wohlbefinden von genitalen vs. nichtgenitalen, fazialen vs. nichtfazialen und Hand‐ vs. Nicht‐Hand‐Beteiligungen zu vergleichen, wurden univariate Kovarianzanalysen (ANCOVA) für den DLQI und WHO‐5 durchgeführt, einschließlich Alter, Geschlecht, BSA, Begleiterkrankungen und Gesichtsbeteiligung (nur für den genitalen Vergleich) als Kovariaten.^10^ Für die Vergleichsanalysen wurden Effektstärken berichtet, wobei ŋ^2^_p_ ≥ 0,01, ŋ^2^_p_ ≥ 0,06 und ŋ^2^_p_ ≥ 0,14 als kleine, mittlere beziehungsweise große Effekte berücksichtigt wurden.^11^ Für psychometrische Validierungszwecke wurden Itemverteilungsmerkmale wie Mittelwert (M), Standardabweichung (SD), Prozentsatz der Items am unteren (Bodeneffekt) und oberen Ende (Deckeneffekte) und Schiefe analysiert. Für die interne Konsistenz wurde Cronbachs Alpha berechnet. Werte > 0,70 wurden als akzeptabel interpretiert.^12^ Um die konvergente Validität zu testen, wurde der WHO‐5 mit dem DLQI (Pearson's Correlation Coefficient) korreliert. In Übereinstimmung mit Weber & Lamb (1970)^13^ wurden Werte zwischen r = 0,36 und r = 0,67 als moderat interpretiert. Werte zwischen r = 0,68 bis r = 0,90 deuten auf hohe Korrelationen hin und Werte über 0,90 deuten auf sehr hohe Korrelationen hin.

## ERGEBNISSE

### Alter, Geschlecht und klinische Merkmale der Patienten

Von insgesamt n = 293 Personen, an welche Fragebögen geschickt wurden, retournierten n = 110 die Fragebögen. Die vollständige Demographie der Stichprobenpopulation ist in Tabelle 1 dargestellt. Die Mehrzahl der Patienten hatte eine NSV (95,5%; n = 105). Von der Patientenkohorte waren 54,5 % Frauen. Das Durchschnittsalter der Probanden betrug 47,45 (SD = 14,65, Median = 49, Bereich: 18–84 Jahre) und die Mehrheit der Patienten (60,0%, n = 66) wies den Hautphototyp III auf. Eine Beteiligung des Gesichtes lag bei 84,5 % [n = 93] und eine der Hände bei 72,7 % [n = 80] vor. Eine Beteiligung des Genitalbereichs wurde bei 47,3% (n = 52) festgestellt. Bei 78,2 % der Patienten bestand eine gleichzeitige Beteiligung genitaler und sichtbarer Bereiche. Bei 67,3 % (n = 74) der Probanden wurde die betroffene BSA dokumentiert. Das mittlere Ausmaß der Erkrankung bei den Patienten mit dokumentierter BSA betrug 13,6 % (SD 17,1). Bei 45,9 % [n = 50] der Patienten lag eine ausgedehnte Vitiligo mit einer BSA > 6,45 % vor. Die mittlere Krankheitsdauer betrug 12,36 Jahre (SD = 11,62, Median = 7,5, Bereich: 1–48 Jahre). Die Gruppen der Patienten mit und ohne genitale Beteiligung waren überwiegend homogen. In Bezug auf Alter und Geschlecht wurde bei männlichen Patienten jedoch häufiger eine genitale Vitiligo diagnostiziert als bei weiblichen Patienten. Darüber hinaus wiesen Patienten ohne genitale Beteiligung häufiger Komorbiditäten auf als Patienten mit genitaler Beteiligung. Patienten mit genitaler Beteiligung wiesen zudem signifikant häufiger eine Gesichtsbeteiligung auf.

**TABELLE 1:** Geschlecht, Alter und klinische Charakteristika der Patienten mit Vitiligo mit und ohne Genitalbeteiligung.

		Gesamt	Genitalbeteiligung	Ohne Genitalbeteiligung	X^2^/ t	p
***Geschlecht, Alter (n)***		110				
Geschlecht, n (%)	Männlich	50 (45,5)	29 (55,8%)	21 (36,2%)	4,23	0,055
	Weiblich	60 (54,5)	23 (44,2%)	37 (63,8%)		
Alter, M ± SD		47,45 (14,65)	48,00 ± 13,40	46,97 ± 15,79	0,368	0,713
***Klinische Charakteristika***						
Art der Vitiligo, n (%)	Nicht‐Segmentale Vitiligo	105 (95,5)	49 (94,2%)	56 (96,6%)	0,34	0,666
	Segmentale Vitiligo	5 (4,5)	3 (5,8%)	2 (3,4%)		
%BSA, M ± SD		13,59 (17,10)	13,00 ± 19,47	14,10 ± 15,03	−0,274	0,785
	Fehlend, n (%)	36 (32,7)	18 (34,6%)	18 (31,0%)		
Krankheitsdauer (Jahre), M ± SD		12,36 (11,62)	12,56 ± 11,17	12,14 ± 12,13	0,133	0,895
	Fehlend, n (%)	14 (12,7)	8 (50,0%)	8 (50,0%)		
Hauttyp, n (%)	Typ I	0 (0%)	0 (0%)	0 (0%)	1,93	0,381
	Typ II	35 (31,8)	16 (30,8%)	19 (32,8%)		
	Typ III	66 (60,0)	29 (55,86%)	37 (63,8%)		
	Typ IV	7 (6,4)	5 (9,6%)	2 (3,4%)		
	Typ V	0 (0%)	0 (0%)	0 (0%)		
	Typ VI	0 (0%)	0 (0%)	0 (0%)		
	Fehlend	2 (1,8)	2 (3,8)			
Komorbidität, n (%) ja		46 (41,8)	15 (28,8%)	31 (53,4%)	6,82	0,012
Nein		64 (58,2)	37 (71,2%)	27 (46,6%)		
Betroffene Körperareale	Gesicht (ja), n (%)	93 (84,5)	49 (52,6%)	44 (47,4%)	7,080	0,009
	Hände (ja), n (%)	80 (72,7)	37 (46,3%)	43 (53,8%)	0,123	0,831

*Abk*.: M, Mittelwert; SD, Standardabweichung; n, Anzahl; BSA, body surface area

### Patientenberichtete Ergebnisse zu Lebensqualität und Wohlbefinden

Der mittlere WHO‐5‐Score lag bei 13,29 (SD = 5,40) und der mittlere DLQI‐Score bei 7,40 (SD = 6,33). Dies deutet auf eine größere Beeinträchtigung des Wohlbefindens als der Lebensqualität hin (Tabellen 2, 3). Die deskriptiven Statistiken für WHO‐5 und DLQI sind in den Tabellen 3 und 4 dargestellt. Vorläufige Korrelationsanalysen zeigten, dass der WHO‐5 negativ mit dem Alter korrelierte (r = –0,281; p = 0,003). Darüber hinaus korrelierte der DLQI moderat mit dem Alter (r = 0,402; p ≤ 0,001) und dem BSA (r = 0,312; 0,003).

**TABELLE 2:** Kovarianzanalyse zu Wohlbefinden und Lebensqualität zwischen Patienten mit und ohne Genital‐, Gesichts‐ und Handbeteiligung.

	GenitalbeteiIigung		Ohne Genitalbeteiligung					
	*n*	*M ± SD*	*n*	*M ± SD*	*F*	*df*	*p*	*ŋ^2^_p_*
Gesundheitsbezogene Lebensqualität (DLQI)	33	7,00 ± 5,45	40	7,48 ± 5,65	0,242	1 66	0,624	0,004
Wohlbefinden (WHO‐5)	52	13,02 ± 5,39	58	13,59 ± 5,41	0,481	1 104	0,490	0,005

	**Gesichtsbeteiligung**		**Ohne Gesichtsbeteiligung**					
	***n***	***M ± SD***	***n***	***M ± SD***	***F***	***df***	***p***	***ŋ^2^_p_***
Gesundheitsbezogene Lebensqualität (DLQI)	62	7,45 ± 5,67	11	6,18 ± 4,90	1,406	1 66	0,270	0,021
Wohlbefinden (WHO‐5)	93	13,23 ± 5,29	17	13,82 ± 6,02	0,277	1 104	0,600	0,003

	**Handbeteiligung**		**Ohne Handbeteiligung**					
	***n***	***M ± SD***	***n***	***M ± SD***	***F***	***df***	***p***	***ŋ^2^_p_***
Gesundheitsbezogene Lebensqualität (DLQI)	52	7,63 ± 6,19	21	6,80 ± 6,75	0,344	1 67	0,559	0,005
Wohlbefinden (WHO‐5)	80	13,11 ± 5,13	30	13,87 ± 6,07	0,116	1 105	0,734	0,001

*Abk*.: DLQI, Dermatology Life Quality Index (Wertebereich 0–30); WHO‐5, Well‐Being‐Index (Wertebereich 0–25); SD, Standardabweichung; ηp^2^, partielles Eta‐Quadrat

*Anmerkung*: Effektstärken sind für vergleichende Analysen berichtet; ηp^2^ ≥ 0,01, ηp^2^ ≥ 0,06 und ηp^2^ ≥ 0,14 wurden als kleine, mittlere und große Effekte betrachtet.

**TABELLE 3:** Deskriptive Statistik von WHO‐5 und DLQI.

	WHO‐5	Item 1:	Item 2:	Item 3:	Item 4:	Item 5:	DLQI
	Gesamtwert Baseline (0–25)	fröhlich und gut gelaunt	ruhig und entspannt	aktiv und voller Energie	frisch und ausgeruht	interessiert	Gesamtwert (0–30)
**M**	13,3	2,9	2,6	2,6	2,4	2,9	7,4
**SD**	5,4	1,2	1,4	1,2	1,4	1,2	6,3
**Schiefe**	−0,3	−0,4	−0,3	−0,2	−0,1	−0,4	1,0
**Kurtosis**	−0,7	−0,8	−0,9	−0,7	−0,9	−0,7	0,5
**Min**	2	0	0	0	0	0	0
**Max**	25	5	5	5	5	5	27

WHO‐5 (0 = kein Wohlbefinden bis 25 = maximales Wohlbefinden); DLQI (0 = keine Beeinträchtigung bis 30 = maximale Beeinträchtigung).

*Abk*.: M, Mittelwert; SD, Standardabweichung

**TABELLE 4:** Distributionscharakteristika des WHO‐5‐Fragebogens.

	WHO‐5 Item 1	WHO‐5 Item 2	WHO‐5 Item3	WHO‐5 Item 4	WHO‐5 Item 5
Zu keinem Zeitpunkt	3 (2,8%)	8 (7,3%)	5 (4,6%)	13 (11,9%)	2 (1,8%)
Ab und Zu	16 (14,7%)	21 (19,3%)	16 (14,7%)	20 (18,3%)	18 (16,5%)
Etwas weniger als die Hälfte der Zeit	22 (20,2%)	15 (13,8%)	31 (28,4%)	20 (18,3%)	15 (13,8%)
Etwas mehr als die Hälfte der Zeit	25 (22,9%)	34 (31,2%)	28 (25,7%)	29 (26,6%)	36 (33,0%)
Meistens	39 (35,8%)	26 (23,9%)	27 (24,8%)	22 (20,2%)	31 (28,4%)
Die ganze Zeit	4 (3,6%)	5 (4,5%)	2 (1,8%)	2 (4,7%)	7 (6.5%)

WHO‐5 (0 = kein Wohlbefinden bis 25 = maximales Wohlbefinden).

**ABBILDUNG 1:**
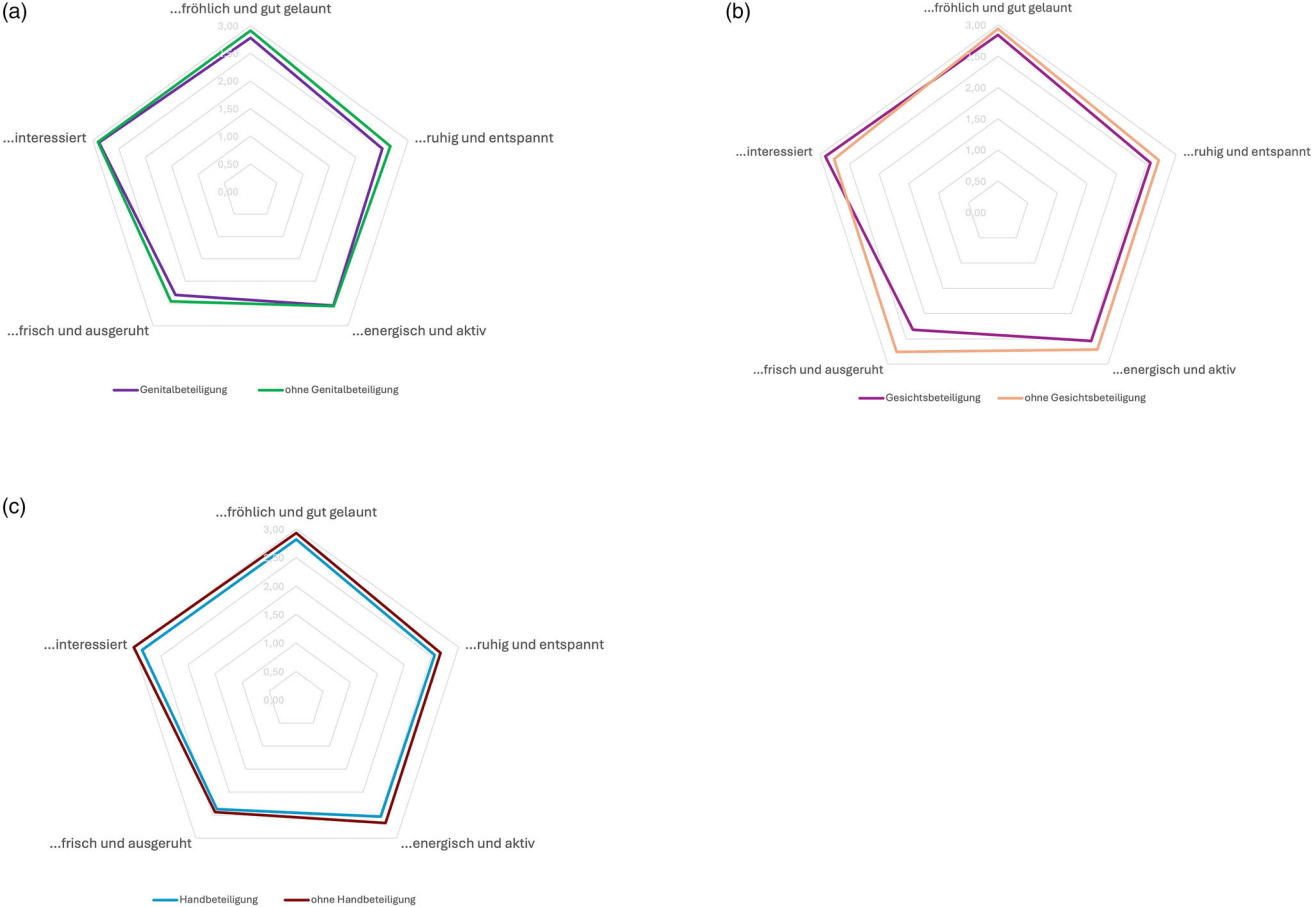
WHO‐5‐Kriterien nach genitaler, Gesichts‐ und Handbeteiligung (ja/nein).

Vergleichende Analysen zeigten keine signifikanten Unterschiede zwischen dem WHO‐5‐ und DLQI‐Gesamtscore zwischen Patienten mit und ohne Beteiligung von Genital, Gesicht und Händen (Tabelle 2). Bei den einzelnen WHO‐5‐Items zeigte sich ein ähnliches Muster zwischen den Gruppen (Abbildung 1).

### Psychometrische Eigenschaften

Der WHO‐5‐Fragebogen zeigte eine gute Reliabilität mit Cronbachs alpha > 0,8 (95%‐Konfidenzintervall [KI]: 0,8; 0,9), was die interne Konsistenz bestätigt. Signifikante moderate Korrelation mit einem anderen von Patienten berichteten Instrument, dem DLQI, wurden mit r = –0,408 (*p* < 0,01) festgestellt, was die konvergente Validität bestätigt.

## DISKUSSION

In dieser Pilotstudie haben wir – unseres Wissens erstmals – das gesundheitsbezogene Wohlbefinden bei deutschen Patienten mit Vitiligo anhand des WHO‐5‐Fragebogens erfasst. Zahlreiche internationale Untersuchungen zeigen, dass Vitiligo mit einer signifikanten Krankheitslast verbunden ist, und viele Studien belegen eine Beeinträchtigung der Lebensqualität.^3^, ^6^

In der Mehrzahl dieser Erhebungen wurde der DLQI als PROM eingesetzt.^14^ In den beiden Phase‐III‐Studien, die zur Zulassung von topischem Ruxolitinib als neuer Behandlungsoption für Patienten mit nicht‐segmentaler Vitiligo (NSV) führten,^15^ zeigte der DLQI im Gegensatz zu anderen PROMs, zum Beispiel der Vitiligo‐Wahrnehmbarkeitsskala,^14^ keine signifikanten Veränderungen der Lebensqualität während dieser Behandlung. Der Grund dafür könnte in der mangelnden Spezifität des DLQIs im Gegensatz zum VitiQoL liegen.^16^ Der VitiQoL ist jedoch derzeit nicht in deutscher Sprache frei verfügbar, was seine Verwendbarkeit in der dermatologischen Routine beeinträchtigt. Derzeit gibt es noch keinen Konsens über die optimale Verwendung verfügbarer PROMs bei Vitiligo, um Stigmatisierung, Wohlbefinden, QoL‐Beeinträchtigung, Krankheitslast, Schweregrad und Auswirkungen der Erkrankung sowie psychosoziale Komorbidität wie Depression und Angststörung bei Vitiligo zu erfassen. Die routinemäßige Anwendung mehrerer PROMs pro Patienten kann informativ, aber teilweise redundant, zeitaufwändig und auch ohne Konsequenzen bleiben, wenn keine Entscheidung über die nachfolgenden interventionellen Schritte getroffen wird, die im Rahmen eines ganzheitlichen therapeutischen Managements der Vitiligo erforderlich sind. In der Stellungnahme der *Vitiligo International Task Force* wurde die Verwendung des VitiQoL vorgeschlagen.^17^ Wir haben kürzlich in einem Positionspapier auf die dringende Notwendigkeit eines Screenings auf psychosoziale Komorbidität bei Patienten mit Vitiligo hingewiesen.^3^ Der WHO‐5‐Fragebogen ist genau wie der DLQI nicht Vitiligo‐spezifisch. Er ist jedoch weniger zeitaufwändig als der DLQI (5 versus 10 Fragen) und kann als frei verfügbares und einfach zu bedienendes erstes Screening‐Instrument für Patienten mit Vitiligo dienen, bevor spezifischere Instrumente wie der Patient Health Questionnaire‐2 (PHQ‐2)^18^ oder die Generalized Anxiety Disorder‐2 (GAD‐2)^19^ verwendet werden. Gemeinsam mit dem *Berufsverband der Deutschen Dermatologen* (BVDD) haben wir den WHO‐5‐Fragebogen als mögliches PROM in einer Dokumentationscheckliste vorgeschlagen, die für Dermatologen bei der Behandlung von Patienten mit Vitiligo vor der Verschreibung von Januskinase‐Hemmern nützlich sein kann.^20^

Obwohl es sich um eine monozentrische Pilotstudie mit einer begrenzten Anzahl von Patienten handelt, stimmen die Verteilung der Subtypen der Vitiligo (NSV vs. SV) und das Geschlechterverhältnis mit den zuvor berichteten Studien überein.^2^, ^21^ Dementsprechend sind 5%–16% der gesamten Vitiligo‐Fälle segmental.^22^, ^23^ Das Durchschnittsalter unserer Patientenkohorte war jedoch höher als das berichtete Inzidenzmaximum von Vitiligo bei Erwachsenen, welches bei 34 Jahren liegt.^24^ Da zumindest in einigen Studien berichtet wurde, dass sozialer Status wie der Familienstand die Beeinträchtigung der Lebensqualität beeinflusst,^14^ können wir davon ausgehen, dass das tatsächliche Wohlbefinden in einer repräsentativeren Patientenkohorte noch geringer ist. Ungeachtet dieser Einschränkungen ergab unsere Studie, dass 14,68% (n = 16) unserer Patienten WHO‐5‐Werte von < 7 aufweisen, was auf eine hohe Wahrscheinlichkeit für eine klinische Depression hinweist. Zur Prävalenz von Depressionen bei Patienten mit Vitiligo in Deutschland liegen derzeit keine aktuellen Daten vor. Je nach verwendeter Methodik lag die weltweite Häufigkeit von Depressionen und depressiven Störungen zwischen 0,1% und 62,3%.^3^, ^6^

Der WHO‐5‐Mittelwert lag bei 13,29. Demnach sind die Beeinträchtigungen des Wohlbefindens bei Vitiligo vergleichbar mit denen bei anderen chronischen Hauterkrankungen wie Psoriasis (M = 15,6)^25^ und Typ‐2‐Diabetes (M = 12,6)^26^ aber auch mit Tumorentitäten wie dem Brustkrebs (M = 13,3).^27^ In unserer Pilotstudie ließ sich kein Zusammenhang zwischen der Beeinträchtigung des Wohlbefindens und den von Vitiligo betroffenen Hautarealen feststellen – weder bei sichtbaren Bereichen wie Gesicht und Händen noch bei nicht sichtbaren wie dem Genitalbereich. Die meisten Patienten hatten eine Beteiligung des Gesichts und der Hände. Ein möglicher Grund hierfür könnte die insgesamt geringe Anzahl an Patienten in unserer Kohorte sein, bei denen keine Beteiligung von Gesicht oder Händen vorlag. Frühere Studien zeigten darüber hinaus keinen konsistenten Einfluss der Beteiligung des Genitalbereichs auf die Lebensqualität bei Patienten mit Vitiligo.^14^ Ebenso war ein dunklerer Hauttyp sowie das Ausmaß der Erkrankung statistisch nicht mit einer größeren Beeinträchtigung des Wohlbefindens unserer Patienten mit Vitiligo korreliert, was höchstwahrscheinlich auf die geringe Anzahl von Patienten mit Hautphototypen höher als III (6,42%, n = 7) zurückzuführen ist. Interessanterweise wurden weder für genitale vs. nichtgenitale noch für faziale‐ vs. Nichtfaziale oder Hand‐ vs. Nicht‐Hand‐Beteiligung signifikante Unterschiede in den WHO‐5‐ und DLQI‐Gesamtwerten gefunden. Dies weist auf eine Beeinträchtigung des Wohlbefindens und der Lebensqualität unabhängig von der betroffenen Körperregion hin. Mit Ausnahme des DLQI bei Patienten mit und ohne Gesichtsbeteiligung berichteten jedoch alle Patienten mit Beteiligung von Gesicht, Genitalbereich oder Händen über ein geringeres Wohlbefinden und stärkere Einschränkungen der Lebensqualität als Patienten ohne entsprechende Beteiligung – auch wenn die Unterschiede statistisch nicht signifikant waren. Unsere Ergebnisse können aufgrund der geringen Stichprobengröße in Untergruppen in Bezug auf die Aussagekraft begrenzt sein. Daher muss der Zusammenhang zwischen bestimmten sichtbaren (Gesicht, Hand) oder genital betroffenen Hautarealen und dem Wohlbefinden in größeren und idealerweise longitudinalen Studien untersucht werden.

Das sekundäre Ziel der vorliegenden Studie war es, die psychometrischen Charakteristika des WHO‐5‐Fragebogens für den Einsatz bei Menschen mit Vitiligo zu untersuchen. Nach der Definition der WHO ist das wesentliche Merkmal von Gesundheit ein Zustand des vollständigen Wohlbefindens. Um diese Ziele zu erreichen, muss ein validiertes Instrument zur Messung des gesundheitsbezogenen Wohlbefindens eingesetzt werden. Versorgungsstudien in vielen Indikationen zeigen, dass der WHO‐5‐Fragebogen positive Eigenschaften in Bezug auf seine Validität und Durchführbarkeit aufweist. In Studien mit älteren Probanden sowie bei psychischen Erkrankungen, Diabetes und neurologischen Erkrankungen hat sich die WHO‐5 als valide erwiesen.^28^, ^29^, ^30^, ^31^ Insbesondere wurde die Validität im Depressionsscreening nachgewiesen, und eine systematische Übersichtsarbeit zeigt ein breites Anwendungsspektrum in der Medizin auf. Aufgrund seiner geringen Anzahl an Fragen und seiner einfachen Scoring‐Methode eignet sich der WHO‐5‐Fragebogen besonders für die Routineversorgung.

Die erste dermatologische Anwendung des WHO‐5‐Fragebogens wurde bei Akne vulgaris in den frühen 80er Jahren veröffentlicht und bezog sich auf die potenzielle Funktion als Screening‐Instrument für Depressionen.^32^ Kürzlich wurden psychometrische Eigenschaften des WHO‐5 bei Psoriasis nachgewiesen.^25^ Unsere Pilotstudie zeigte positive Eigenschaften für die Anwendung des WHO‐5‐Fragebogens bei Patienten mit Vitiligo. Die Ergebnisse deuten darauf hin, dass der WHO‐5‐Fragebogen zur Erfassung des Wohlbefindens der Patienten in der Vitiligo‐Routineversorgung beitragen kann, da der Fragebogen eine gute Reliabilität und Validität zeigte.

Unsere Studie hat jedoch Einschränkungen und Schwächen. Erstens war die Anzahl der Patienten, die den Fragebogen beantworteten, begrenzt und umfasste nur Patienten, die in einem klinischen Zentrum in Deutschland behandelt wurden. Zweitens könnten die Ein‐ und Ausschlusskriterien (siehe Abschnitt Patienten und Methodik) zu einer Verzerrung geführt haben. Darüber hinaus können Alter, Geschlecht und klinische Merkmale der Patienten, die nicht auf unseren Fragebogen geantwortet haben, unterschiedlich sein und damit auch ihre DLQI‐ und WHO‐5‐Werte. Die Untersuchung des Einflusses von Vitiligo auf das Wohlbefinden in größeren Kohorten deutscher Patienten unter Verwendung von Kernergebnissen, wie sie von der *Vitiligo International Task Force for an Agreed List of Core Dat*a (VITAL)^33^ vorgeschlagen wurden, könnte unsere Ergebnisse bestätigen und erweitern. Zukünftige Studien sollten auch die Veränderungen des Wohlbefindens bei Patienten mit Vitiligo als Reaktion auf die Behandlung untersuchen.

## DANKSAGUNG

Open access Veröffentlichung ermöglicht und organisiert durch Projekt DEAL.

## INTERESSENKONFLIKT

M.B. erhielt Honorare von AbbVie, CME‐Welt, Incyte, MSD und Pfizer (Unternehmen, die an der Entwicklung von Medikamenten für Vitiligo beteiligt sind) für beratende Funktionen. Außerdem erhielt er Honorare von AbbVie, CME‐Welt, derCampus, Incyte, Isispharma, Janssen‐Cilag, Omnicuris, Pfizer und StreamedUp für wissenschaftliche Vorträge zum Thema Vitiligo. Er ist Sub‐Investigator oder Deputy Principal Investigator in klinischen Studien von AbbVie, Incyte, MSD und Pfizer an Patienten mit Vitiligo. Zudem erhält er Forschungsgelder von Incyte (VitiligoHealth/EU‐DE‐D‐24053). R.S. erhielt Vortrags‐, Forschungs‐ und Beratungshonorare und/oder Reisekosten von AbbVie, Almirall, Amgen, Beiersdorf AG, CME‐Welt, EPG‐Health, Incyte, Janssen‐Cilag, Leo Pharma, Novartis und UCB. K.S. und J.O. haben keine Interessenkonflikte.

## References

[1] Bibeau K , Pandya AG , Ezzedine K , et al. Vitiligo prevalence and quality of life among adults in Europe, Japan and the USA. J. Eur. Acad. Dermatol. Venereol.. 2022;36:183‐1844. 10.1111/jdv.18257 PMC9544885 35611638

[2] Böhm M , Tanew A . CME Article: Vitiligo. J. Dtsch. Dermatol. Ges.. 2025;23:968‐987. 40788636 10.1111/ddg.15706 PMC12338405

[3] Böhm M , Sommer R , Gieler U , et al. Vitiligo – a disease: A position paper on stigmatization, life quality impairment and psychosocial comorbidity. J. Dtsch. Dermatol. Ges.. 2024;22:1327‐1333. 39167551 10.1111/ddg.15503

[4] Krüger C , Schallreuter KU . Stigmatisation, Avoidance Behaviour and Difficulties in Coping are Common Among Adult Patients with Vitiligo. Acta. Derm. Venereol.. 2015;95:553‐558. 25269389 10.2340/00015555-1981

[5] Radtke MA , Schäfer I , Gajur A , et al. Willingness‐to‐pay and quality of life in patients with vitiligo. Br. J. Dermatol.. 2009;161:134‐139. 19298268 10.1111/j.1365-2133.2009.09091.x

[6] Ezzedine K , Eleftheriadou V , Jones H , et al. Psychosocial Effects of Vitiligo: A Systematic Literature Review. Am. J. Clin. Dermatol.. 2021;22:757‐774. 34554406 10.1007/s40257-021-00631-6 PMC8566637

[7] Allgaier AK , Liwowsky I , Kramer D , et al. Der WHO‐5‐Fragebogen zum Wohlbefinden als Screeninginstrument für Depression bei Altenheimbewohnern [Screening for depression in nursing homes: validity of the WHO (Five) Well‐Being Index]. Neuropsychiatr. 2011;25:208‐215. 22136943

[8] Finlay AY , Khan GK . Dermatology Life Quality Index (DLQI) – a simple practical measure for routine clinical use. Clin. Exp. Dermatol.. 1994;19:210‐216. 8033378 10.1111/j.1365-2230.1994.tb01167.x

[9] Hongbo Y , Thomas CL , Harrison MA , et al. Translating the science of quality of life into practice: what do dermatology life quality index scores mean? J. Invest. Dermatol.. 2005;125:659‐664. 16185263 10.1111/j.0022-202X.2005.23621.x

[10] Raab GM , Day S , Sales J . How to select covariates to include in the analysis of a clinical trial. Control Clin. Trials. 2000;21:330‐342. 10913808 10.1016/s0197-2456(00)00061-1

[11] Cohen J . Statistical power Analysis for the Behavioral Sciences. 2. ed., Erlbaum, 1988.

[12] Cronbach LJ . Coefficient alpha and the internal structure of tests. Psychometrica. 1951;16:297‐334.

[13] Weber J , Lamb D . Statistics and Research in Physical Education. St. Louis: CV, Mosby Co, 1970.

[14] Chernyshov PV , Tomas‐Aragones L , Manolache L , et al. Quality of life measurement in vitiligo. Position statement of the European Academy of Dermatology and Venereology Task Force on Quality of Life and Patient Oriented Outcomes with external experts. J. Eur. Acad. Dermatol. Venereol.. 2023;37:21‐31. 36259656 10.1111/jdv.18593

[15] Rosmarin D , Passeron T , Pandya AG , et al. Two Phase 3, Randomized, Controlled Trials of Ruxolitinib Cream for Vitiligo. N. Engl. J. Med.. 2022;387:1445‐1455. 36260792 10.1056/NEJMoa2118828

[16] Lilly E , Lu PD , Borovicka JH , et al. Development and validation of a vitiligo‐specific quality‐of‐life instrument (VitiQoL). J. Am. Acad. Dermatol.. 2013;69:e11‐e18. 22365883 10.1016/j.jaad.2012.01.038

[17] van Geel N , Speeckaert R , Taïeb A , et al. Worldwide expert recommendations for the diagnosis and management of vitiligo: Position statement from the International Vitiligo Task Force Part 1: towards a new management algorithm. J. Eur. Acad. Dermatol. Venereol.. 2023;37:2173‐2184. 37746876 10.1111/jdv.19451

[18] Kroenke K , Spitzer RL , Williams JB . The Patient Health Questionnaire‐2: Validity of a Two‐Item Depression Screener. Medical Care. 2003;41:1284‐1292. 14583691 10.1097/01.MLR.0000093487.78664.3C

[19] Kroenke K , Spitzer RL , Williams JB , et al. Anxiety disorders in primary care: prevalence, impairment, comorbidity, and detection. Ann. Intern. Med.. 2007;146:317‐325. 17339617 10.7326/0003-4819-146-5-200703060-00004

[20] Augustin M , Böhm, M , Berneburg M , et al. Vitiligo: Krankheitslast erfordert medizinische Versorgung. Berufspolitik. 2024;72:20‐25.

[21] Bergqvist C , Ezzedine K . Vitiligo. A review. Dermatology. 2020;236:571‐592. 32155629 10.1159/000506103

[22] Hann SK , Lee HJ . Segmental vitiligo: clinical findings in 208 patients. J. Am. Acad. Dermatol.. 1996;35:671‐674. 8912558 10.1016/s0190-9622(96)90718-5

[23] Silverberg NB . Update on childhood vitiligo. Curr. Opin. Pediatr.. 2010;22:445‐452. 20616733 10.1097/MOP.0b013e32833b6ac3

[24] Jin Y , Roberts GHL , Ferrara TM , et al. Early‐onset autoimmune vitiligo associated with an enhancer variant haplotype that upregulates class II HLA expression. Nat. Commun.. 2019;10:391. 30674883 10.1038/s41467-019-08337-4 PMC6344500

[25] Sommer R , Mrowietz U , et al. Measuring well‐being in psoriasis: psychometric properties of the WHO‐5 questionnaire. J. Eur. Acad. Dermatol. Venereol.. 2022;36:e986‐e987. 35788987 10.1111/jdv.18396

[26] Pintaudi B , Lucisano G , Gentile S , et al. Correlates of diabetes‐related distress in type 2 diabetes: Findings from the benchmarking network for clinical and humanistic outcomes in diabetes (BENCH‐D) study. J. Psychosom. Res.. 2015;79:348‐354. 26526307 10.1016/j.jpsychores.2015.08.010

[27] Hoffman CJ , Ersser SJ , Hopkinson JB , et al. Effectiveness of mindfulness‐based stress reduction in mood, breast‐ and endocrine‐related quality of life, and well‐being in stage 0 to III breast cancer: a randomized, controlled trial. J. Clin. Oncol.. 2012;30:1335‐1342. 22430268 10.1200/JCO.2010.34.0331

[28] Bonsignore M , Barkow K , Jessen F , Heun R . Validity of the five‐item WHO Well‐Being Index (WHO‐5) in an elderly population. Eur. Arch. Psychiatry Clin. Neurosci.. 2001;251(Suppl 2):II27–31. 10.1007/BF03035123 11824831

[29] Mergl R , Seidscheck I , Allgaier AK , et al. Depressive, anxiety, and somatoform disorders in primary care: prevalence and recognition. Depress Anxiety.. 2007;24:185‐195. 16900465 10.1002/da.20192

[30] De Wit M , Pouwer F , Gemke RJ , et al. Validation of the WHO‐5 Well‐Being Index in adolescents with type 1 diabetes. Diabetes Care. 2007;30:2003‐2006. 17475940 10.2337/dc07-0447

[31] Schneider CB , Pilhatsch M , Rifati M , et al. Utility of the WHO‐Five Well‐being Index as a screening tool for depression in Parkinson's disease. Mov. Disord.. 2010;25:777‐783. 20108365 10.1002/mds.22985

[32] Henkel V , Moehrenschlager M , Hegerl U , et al. Screening for depression in adult acne vulgaris patients: tools for the dermatologist. J. Cosmet. Dermatol.. 2002;1:202‐207. 17147540 10.1111/j.1473-2165.2002.00057.x

[33] van Geel N , Hamzavi IH , Pandya AG , et al. Vitiligo International Task force for an Agreed List of core data (VITAL): study protocol of a vitiligo core outcome set (COS) and contextual factors for clinical trials, registries, and clinical practice. Trials. 2022;23:591. 35871019 10.1186/s13063-022-06497-1 PMC9308182

